# The bromodomain protein Bdf3 is sufficient to activate expression of a procyclin gene in bloodstream stage African trypanosomes

**DOI:** 10.1128/msphere.00021-26

**Published:** 2026-06-01

**Authors:** Evan J. Kim, Ethan L. Goroza, Ashley Y. Tan, Jolyne J. Lin, Kieran C. Saucedo, Fuminori Tanizawa, Jaclyn E. Smith, Danae Schulz

**Affiliations:** 1 Department of Biology, Harvey Mudd College 173209, Claremont, California, USA; 2 Institute for Genome Sciences, University of Maryland, Baltimore, Maryland, USA; Cleveland State University, Cleveland, Ohio, USA

**Keywords:** chromatin, *Trypanosoma brucei*, epigenetics, bromodomain protein, molecular parasitology

## Abstract

The early-branching eukaryotic parasite, *Trypanosoma brucei*, lives extracellularly in the mammalian host, where it expresses variant surface glycoprotein (VSG). Antigenic variation of VSGs allows the parasite to evade the host immune response while in the bloodstream. Upon transition to the tsetse fly vector, the parasite remodels its surface to express procyclin protein from a limited set of *EP* and *GPEET* genes. While the environmental signals that trigger the switch from expressing *VSG* genes to expressing *EP* and *GPEET* genes are known, the molecular mechanism that initiates transcription of the *EP* and *GPEET* genes during parasite differentiation to the procyclic stage within the tsetse is not well understood. Previous work has shown that the chromatin-interacting bromodomain protein Bdf3 is absent from the RNA polymerase I promoter of the *EP* locus in bloodstream parasites, but appears there shortly after initiation of differentiation from the bloodstream to the procyclic stage. Here, we show that tethering of Bdf3 to the *EP* promoter using a dCas9-Bdf3 fusion protein and a guide RNA is sufficient to increase transcript levels of *EP1* in bloodstream parasites, where the gene is normally silenced. This result supports the model that Bdf3 appears at the *EP* locus during differentiation to facilitate initiation of transcription, which is consistent with the role of Bdf3 as a transcriptional activator in other systems. Understanding gene regulatory mechanisms in this early-branching eukaryote may help build a more complete picture of the conserved and unique features of gene regulatory proteins across diverse biological systems.

**IMPORTANCE:**

The *Trypanosoma brucei* parasite is transmitted via the tsetse fly vector to a mammalian host , where it causes African trypanosomiasis, a fatal disease that imposes a severe human and economic burden for people living in areas of sub-Saharan Africa. While drug treatments have improved for some strains, some infections still require treatment with drugs that have serious side effects. One avenue for drug development is to try to manipulate the life cycle of the parasite to make it poorly adapted to the mammalian host. However, this requires detailed knowledge of the mechanisms by which parasites regulate their genes as they progress through the life cycle. Here, we show that the DNA-interacting bromodomain protein Bdf3 may be a key driver for turning on insect-specific genes that code for insect-stage surface proteins. This finding could aid in the development of life cycle-manipulating drugs and shed light on how gene regulatory mechanisms evolved.

## INTRODUCTION

Numerous parasites transition between hosts as part of their natural life cycle. Because host environments can differ dramatically with respect to temperature, pH, and nutrient availability, parasites that transition between hosts must evolve systems to adapt to differing environments. One such parasite is the African trypanosome, *Trypanosoma brucei. T. brucei* spp. are unicellular, eukaryotic, protozoan parasites that infect humans and ungulates and cause human and animal African trypanosomiasis disease. Untreated African trypanosomiasis is usually fatal and imposes a severe human and economic burden for people living in sub-Saharan Africa, where the disease is endemic (1, 2). *T. brucei* cycle between a tsetse fly vector and the bloodstream of a mammalian host. Compared to the tsetse, the bloodstream environment of the mammalian host is warmer and glucose-rich, and the extracellular parasites must contend with a robust antibody response mounted by the host immune system. The parasite has evolved to live in the mammalian bloodstream by antigenically varying the proteins on its surface, which are coded for by a large repertoire of variant surface glycoprotein (*VSG*) genes (3). Antigenic variation of VSG surface proteins allows bloodstream parasites to successfully evade the host antibody response. Upon transition to the fly midgut following a tsetse bloodmeal, the parasites alter their glucose metabolism (4–7) and express a far more limited repertoire of procyclin surface proteins coded by the *GPEET* and *EP* genes (8). GPEET proteins contain a pentapeptide repeat of the GPEET amino acid sequence, while EP proteins contain a dipeptide repeat (8, 9). *GPEET, EP1, EP2,* and *EP3* are all co-expressed within the first few hours after differentiation to the procyclic form. GPEET protein predominates on the parasite surface early in development, and this protein is replaced with EP isoforms later in development within the midgut (10). Since the tsetse lacks an antibody-based immune response, antigenic variation of *VSG* genes is no longer required, and instead, the GPEET and EP procyclin proteins are thought to shield the parasites from proteases expressed by the tsetse (11). Recent results indicate that parasites with all procyclin genes deleted are able to complete the life cycle within the fly (12). However, they compete poorly with parasites that retain procyclin, indicating that procyclin confers a survival advantage. In contrast, bloodstream parasites do not tolerate perturbation of the VSG coat (13, 14). Thus, remodeling of the parasite surface is important following transition to either the bloodstream or the tsetse environments.

Whether changes in the surface protein coat are sufficient to drive differentiation in *T. brucei* is an intriguing and open question in the field. Amiguet-Vercher et al. observed that *VSG* genes from silent expression sites in bloodstream parasites showed higher levels of transcription as parasites differentiated from the bloodstream to the procyclic form (15). Another study found that derepression of silent *VSG*s was associated with transcriptome changes consistent with parasites transitioning from the bloodstream to the procyclic stage (16). In addition, forced expression of a second VSG in bloodstream parasites caused *T. brucei* parasites to show some signs of developmental progression, including G1-phase dormancy and expression of the protein associated with differentiation gene, *PAD1* (17, 18). Transcript levels of *EP1* increase in stumpy parasites that form as parasites prepare for transition to the fly. However, to our knowledge, it is not yet known if forced expression of procyclin in bloodstream parasites is sufficient to trigger developmental progression.

While it has been established that expression of *GPEET* and *EP* genes is transcriptionally and developmentally regulated (19, 20), the molecular mechanism that initiates transcription of these genes following parasite transition to the fly is poorly understood. Unusually for a eukaryote, the *GPEET* and *EP* procyclin protein coding genes are transcribed by RNA polymerase I (Pol I) (19, 21–23). Pol I is also the polymerase that transcribes *VSG* genes. In addition to the unusual transcription of protein coding genes by Pol I, *Trypanosoma brucei* exhibits additional gene organization and regulatory features that are not found in better-studied model organisms, such as yeast, worms, or flies. African trypanosome genes are organized in polycistronic arrays (24), and mRNA maturation includes co-transcriptional polyadenylation and trans-splicing (reviewed in reference 25). The spliced leader sequence that is spliced onto mRNAs is transcribed from the spliced leader array (26, 27). Interestingly, the transcriptionally active *VSG* locus that is transcribed by Pol I physically associates with the DNA that codes for the spliced leader array in bloodstream parasites (28, 29). The procyclin locus that is transcribed by Pol I has also been found to associate with the spliced leader array at the procyclic stage of the life cycle (29). Unlike bacteria, the genes in polycistronic arrays in *T. brucei* are not functionally related. Trypanosomes lack obvious sequence-specific DNA binding transcription factors, and whether sequence-specific RNA polymerase II (Pol II) promoters exist is somewhat controversial (25, 30). Most protein coding genes in *T. brucei* are transcribed by Pol II in polycistronic units with limited transcriptional regulation (reviewed in reference 25 and recently corroborated with SLAM-seq [31]). In contrast, the loci that encode procyclin *EP* and *GPEET* genes have a well-defined promoter sequence and are transcriptionally regulated (19, 21–23). Thus, the transcriptional regulation of procyclin genes offers a unique opportunity to examine transcriptional regulation in a highly diverged, early branching eukaryote.

Despite the lack of transcriptional control for Pol II genes, *T. brucei* parasites do harbor much of the machinery associated with the histone code that is best characterized in model systems such as yeast, worms, and flies (32). This includes numerous post-translational modifications of histone proteins (33) as well as proteins that read, write, and erase those modifications (16, 34–40). Several specific histones and histone modifications are associated with Pol II transcription start sites, including H2A.Z, H2B.V (41, 42), H4K10ac (34), and H3K4me3 (43). Numerous chromatin-associated readers, writers, and erasers have been localized to Pol II transcription start sites in *T. brucei* (16, 34, 38). The targets of the histone acetyltransferases HAT1, HAT2, and HAT3 have also been identified. HAT3 acetylates H4K4 (36, 44), a mark that is not associated with transcription start sites in *T. brucei*. HAT1 is thought to acetylate the histone variants H2A.Z and H2B.V as well as H4K2 (45), while HAT2 acetylates H4K2, H4K5 (45), and H4K10 (35, 45). However, the molecular mechanisms that control the deposition of histone marks at Pol II transcription start sites are not yet fully understood.

Much less work has been done to characterize exactly which chromatin-interacting proteins bind to Pol I sites. Whether the deposition of histone marks changes during differentiation from bloodstream to insect stages at Pol I or Pol II sites has also not yet been explored. That said, epigenetic regulation of *VSG* genes that are transcribed by Pol I has been documented by several groups. Knockdown of many chromatin-associated proteins leads to loss of monoallelic *VSG* expression in bloodstream parasites (16, 35, 37, 40, 46), and active Pol I-transcribed *VSG* expression sites are depleted of nucleosomes (47, 48). Additionally, the histone acetyltransferase HAT3 is important for telomeric silencing in expression sites controlled by Pol I (36, 49). Thus, epigenetic factors may act as key regulators of genes transcribed by Pol I, including the *EP* and *GPEET* genes.

One of the best-characterized domains found in proteins that act as chromatin readers is the bromodomain, which recognizes acetylated lysines on histone tails and has an established role in gene activation (50–54). In murine models, bromodomain proteins maintain cell identity and help regulate differentiation (55–57). Bromodomain proteins have also been examined in protozoan parasites, where it has been found that inhibition via small molecules or genetic manipulation results in defects in transcription for *Toxoplasma gondii* (58) and *Leishmania mexicana (59*) as well as developmental blocks in *Trypanosoma cruzi* (60, 61) and *Plasmodium falciparum* (62, 63). Small molecule inhibition of bromodomain proteins causes defects in egg development in *Schistosoma japonicum (64*). In *T. brucei*, bromodomain proteins are also involved in differentiation processes, both in bloodstream (16) and in procyclic (65) stages. *T. brucei* contains seven proteins with bromodomains identified through homology (34, 38, 66). Bdf1-6 bind Pol II transcription start sites in bloodstream parasites (16, 34, 38), while Bdf7 localizes to transcription termination regions (38). Staneva et al. classified Bdf1, Bdf3, and Bdf4 as class I transcription start region-associated factors because of the sharp, high peaks observed in ChIP-seq experiments using antibodies against tagged Bdf1, Bdf3, and Bdf4. Based on their genomic distribution, the authors hypothesized that Bdf1, Bdf3, and Bdf4 were likely to be involved in transcription initiation (38). Bdf2, Bdf5, and Bdf6 showed broader distributions, and thus were classified as class II transcription start region-associated factors. Bdf2, Bdf5, and Bdf6 were hypothesized to be involved in transcriptional elongation based on this distribution (38). Bromodomain protein localization at *T. brucei* Pol I transcription start sites is not as well characterized. Both Bdf2 and Bdf3 were found to associate with Pol I bloodstream expression site promoters in bloodstream parasites using ChIP (16), but to our knowledge, the others have not been rigorously tested for binding at Pol I sites. Based on their genomic localization at Pol II transcription start sites and association with transcriptional activation, we hypothesized that bromodomain proteins in *T. brucei* might also be important for initiating transcription of Pol I-driven procyclin genes that are transcriptionally activated as parasites transition from the bloodstream to the insect stage.

Using CUT&RUN (67), we followed the genomic localization of the bromodomain protein Bdf3 in pleomorphic parasites induced to differentiate from the bloodstream to the procyclic stage via treatment with cis-aconitate and incubation at 27°C (68). We found that both the *EP* locus and the *GPEET* locus lacked genomic occupancy of Bdf3 in bloodstream parasites. Three hours after differentiation was induced, we observed *de novo* occupancy of Bdf3 at both the *EP* locus and the *GPEET* locus. Indeed, these were the only loci analyzed where Bdf3 was consistently absent from a genomic site in bloodstream parasites but present following induction of differentiation (69). While the presence of Bdf3 at the *EP* and *GPEET* loci implicates the protein as contributing to initiation of transcription at that locus, its mere presence does not definitively prove its ability to increase transcript levels of procyclin genes. We thus set out to test whether Bdf3 was sufficient to increase transcript levels at the *EP1* locus in bloodstream parasites, where the locus should normally be silenced. We engineered a tethering system inspired by CRISPR activation-based systems developed for other organisms (70, 71) and introduced it into a previously validated reporter system for *EP1* transcript levels (72). This system allowed us to artificially drag Bdf3 to the *EP1* locus in bloodstream parasites, where *EP1* is normally silenced. Here, we show that Bdf3 is sufficient to increase transcript levels of *EP1* under these conditions. This system can be used as a platform to study transcriptional activation for a host of other putative activators.

## RESULTS

### The dCas9-Bdf3 fusion protein is inducibly expressed in *Trypanosoma brucei*

**Fig 1:**
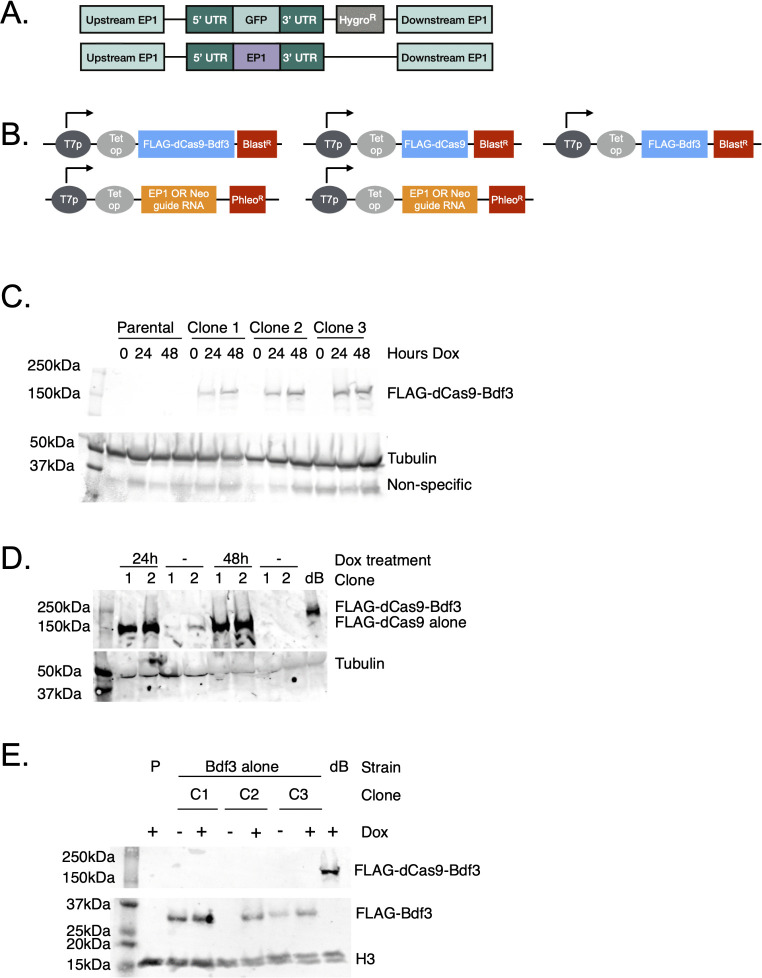
The dCas9-Bdf3 fusion protein and the dCas9 protein are inducibly expressed. (**A**) Schematic of the *EP1/GFP* reporter line construct. Image reproduced from reference 72. (**B**) Schematic of constructs integrated into the *T. brucei* genome in order to inducibly express the FLAG-dCas9-Bdf3 fusion protein, the FLAG-dCas9 protein, or the FLAG-Bdf3 protein. Schematics for the constructs to express guide RNAs targeted to either *EP1* or *NEO* are also shown. T7p, T7 promoter; Tet op, tetracycline operator; Blast^R^, *BSD* gene to generate resistance to blasticidin; Phleo^R^, *BLE* gene encoding resistance to phleomycin. Both constructs are targeted to the rDNA locus. (**C**) Western blot of *T. brucei* extracts transfected with the FLAG-dCas9-Bdf3 fusion construct and harvested at the indicated time points following treatment with 1 µg/mL dox. Three independent clones were analyzed along with a parental control that was not transfected with the FLAG-dCas9-Bdf3 construct (labeled Parental). Blots were probed with anti-FLAG to detect FLAG-dCas9-Bdf3 and anti-tubulin to detect tubulin protein. (**D**) Western blot of *T. brucei* extracts transfected with the FLAG-dCas9-alone construct and harvested at the indicated time points following treatment with 1 µg/mL dox. For each time point, a parasite sample untreated with dox was harvested, indicated by a “−” symbol. −dox samples harvested in parallel with the 24 h dox-treated samples were loaded to the right of 24 h dox-treated extracts, and −dox samples harvested in parallel with the 48 h dox-treated samples were loaded to the right of the 48 h dox-treated extracts. Two independent clones were analyzed, and an extract containing a FLAG-dCas9-Bdf3 construct was used as a positive control (labeled dB). Blots were probed with anti-FLAG to detect FLAG-dCas9 and FLAG-dCas9-Bdf3 and with anti-tubulin to detect tubulin protein as a loading control. Note that FLAG-dCas9-Bdf3 looks like it is migrating higher in this blot due to smiling of the gel, which can also be observed in the tubulin samples. (**E**) Western blot of *T. brucei* extracts transfected with the FLAG-Bdf3 construct and harvested after 48 h of treatment with 1 µg/mL dox. − indicates untreated samples. Three independent clones were analyzed (labeled C1, C2, and C3) along with a parental control that was not transfected with the FLAG-Bdf3 construct. An extract containing a FLAG-dCas9-Bdf3 construct was used as a positive control (labeled dB). Blots were probed with anti-FLAG to detect FLAG-Bdf3 and FLAG-dCas9-Bdf3, and anti-H3 to detect histone H3 as a loading control.

Previous work in our lab showed that Bdf3 is absent from the *EP1* promoter in bloodstream parasites (69). After treatment with cis-aconitate and incubation at 27°C to induce differentiation to the procyclic form, Bdf3 localizes to the *EP* locus at 3 h post-induction and remains there until differentiation is completed at 72 h post-induction (69). However, whether Bdf3 occupancy at the *EP1* locus is sufficient to increase transcript levels of the *EP1* gene is unknown. In order to test whether Bdf3 is sufficient to induce expression of *EP1* in bloodstream parasites, we cloned a construct that would allow us to artificially recruit Bdf3 to the *EP1* locus in *EP1/GFP* reporter bloodstream parasites (Fig. 1A) (72). Because *EP1* transcript levels are influenced by cell density in pleomorphic strains (73), we carried out the study in a monomorph strain to eliminate density-dependent effects on *EP1* transcript levels. To recruit Bdf3 to the *EP1* locus in bloodstream parasites, we fused the entire Bdf3 open reading frame to a catalytically dead version of the Cas9 protein (dCas9) using a flexible GS linker (74). The dCas9 protein is recruited to a specific genomic locus using a guide RNA, but two mutations in the active site prevent dCas9 from creating a double-strand break at that location (70, 75). In model systems, fusion of dCas9 to transcriptional activation domains (CRISPR activation) or transcriptional inhibition domains (CRISPR inhibition), and targeting of these fusion proteins to a gene promoter using a guide RNA, modulates transcription for the target gene (70, 71). CRISPR activation and CRISPR inhibition systems have been successfully adapted in mammalian cells, plants (76), and in the protozoan parasite *Plasmodium falciparum* (77). Excellent work adapting CRISPR-Cas9 systems for genome editing in *T. brucei* (78–80) allowed us to take advantage of previously built constructs to set up the CRISPR activation system. To our knowledge, we are the first to adapt a dCas9-based CRISPR-activation system to *T. brucei*.

To address whether Bdf3 can increase transcript levels at a silent locus, our FLAG-tagged dCas9-Bdf3 fusion construct was cloned into a vector that allows inducible expression using the tetracycline-on system (tet-on) (Fig. 1B) (81). The construct was stably integrated into the genome using homology to the rDNA locus. An additional FLAG-tagged dCas9 construct was integrated to use as a control. Treatment for 1 day or 2 days with doxycycline (dox) showed inducible expression of both the dCas9-Bdf3 fusion protein and the dCas9 protein in transfected parasites, with both proteins appearing at the expected size (Fig. 1C and D). We did not observe background expression of dCas9-Bdf3 in the −dox condition for any of the three clones (Fig. 1C). We observed a robust expression increase in dCas9 protein levels after doxycycline treatment, although a faint band was observed in both clones in uninduced samples (−dox). Thus, we proceeded with clone 1. As an additional control, we cloned the Bdf3 open reading frame into the same vector that was used for both dCas9-Bdf3 and dCas9 alone and tested inducible expression by Western blot (Fig. 1E). We observed the lowest level of background expression in clone 2, as evidenced by undetectable protein in the −dox condition. Further experiments continued with this clone. Once inducible expression was verified for both the dCas9-Bdf3 strain and the dCas9-alone strain, we transfected both strains with guide RNAs targeted to either the *EP1* promoter or a locus unrelated to differentiation. We chose the *NEO* locus to use as the unrelated control. The *NEO* gene was previously used to select for integration of the T7 polymerase and tet repressor (81), which is present in the background of the *EP1/GFP* reporter strain. Guide RNA constructs were designed to target the *EP1* promoter (19, 21, 23, 82) or the *NEO* gene using the Eukaryotic Pathogen CRISPR guide RNA/DNA Design Tool (83). Tet/dox-inducible guide RNA constructs were stably integrated into the trypanosome genome using selection with phleomycin (Fig. 1B), as in reference 78.

### The bromodomain protein Bdf3 is sufficient to increase transcript levels of *EP1/GFP* when targeted to the *EP1* locus

**Fig 2:**
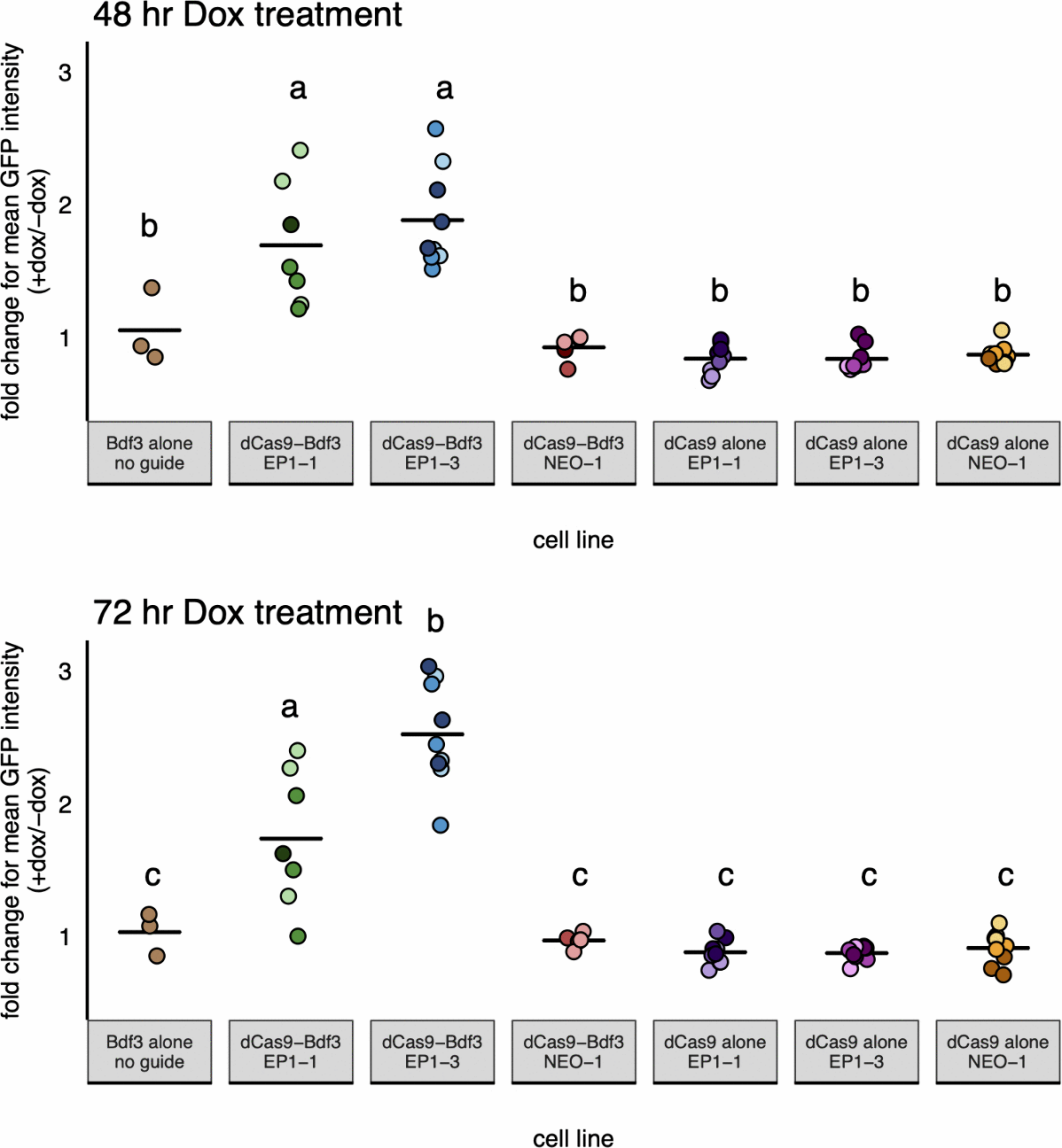
dCas9-Bdf3 targeted to the *EP1* locus is sufficient to increase *EP1/GFP* transcript levels in bloodstream *T. brucei* parasites. Quantification of flow cytometry data processed to compute the fold change in mean fluorescence intensity of parasites treated with dox vs untreated parasites. One microgram per milliliter dox treatment was used to induce expression of dCas9-Bdf3, dCas9 alone, Bdf3 alone, and guide RNAs targeted to either the *EP1* locus (EP1-1 and EP1-3 guides) or the *NEO* locus (NEO-1 guide). Experiments in which parasites were treated for 48 h with dox are shown in the top panel, and those where parasites were treated for 72 h with dox are shown in the bottom panel. Three independent clones were analyzed for each of the dCas9-Bdf3 and dCas9-alone strains. Only one clone of Bdf3 alone was analyzed with three biological replicates. Dots are color-coded such that biological replicates for the same clone are the same color. Data were analyzed using ANOVA followed by a Tukey honestly significant difference (HSD) *post hoc* analysis. Two different ANOVA were performed, one for 48 h and one for 72 h dox treatment. Significant differences between groups are shown with compact letter display. A full set of *P*-values for each comparison is provided in Tables S1 and S2.

To ascertain whether Bdf3 is sufficient to increase transcript levels of *EP1/GFP* in bloodstream parasites, where it should not be highly expressed, we measured *EP1/GFP* levels by flow cytometry in *EP1/GFP* reporter parasites harboring either *EP1* guides or *NEO* guides in both dCas9-Bdf3 strains and dCas9-alone strains. Three different clones were chosen to analyze for each guide RNA tested, and dox was used to induce expression of both the guide RNA and either the dCas9-Bdf3 fusion protein or the dCas9-alone protein. Flow cytometry measurements were taken after 48 h and 72 h of dox treatment. For most clones, additional biological replicates were analyzed, except in the case where a clone was inadvertently lost. For each sample, the mean GFP fluorescence intensity was obtained for the +dox treatment, where dCas9-Bdf3, dCas9 alone, and the guide RNA were induced, and for the uninduced −dox treatment used as the control. The fold change in mean GFP fluorescence intensity for the +dox versus the −dox samples was computed and is shown in Fig. 2. We found that there was a significant increase in +dox/−dox fold changes for mean GFP fluorescence intensity at both time points in parasites with dCas9-Bdf3 targeted to the *EP1* locus using two different guide RNAs (dCas9-Bdf3 EP1-1 and dCas9-Bdf3 EP1-3), when compared to control parasites harboring dCas9-Bdf3 targeted to the *NEO* locus (dCas9-Bdf3 NEO-1) (Fig. 2; Fig. S1; Tables S1 and S2). This result indicates that the increase in *EP1/GFP* expression occurs only when the fusion protein is targeted to the *EP1* locus and is thus locus dependent. We also found that there was a significant increase in +dox/−dox fold changes in mean GFP fluorescence intensity when dCas9-Bdf3 parasites targeted to the *EP1* locus were compared to dCas9-alone parasites targeted to the *EP1* locus at both time points, indicating that dCas9 alone is not sufficient to increase *EP1/GFP* and that Bdf3 is also required (Fig. 2; Tables S1 and S2). We do not have evidence that overexpression of Bdf3 alone is sufficient to increase transcript levels of *EP1/GFP*, as there was no significant difference in mean GFP fluorescence intensity fold changes for Bdf3-alone parasites compared to dCas9-alone parasites targeted to either *NEO1* or *EP1* (Tables S1 and S2). There is no significant difference in +dox/−dox fold changes in dCas9-alone parasites targeted to *EP1* compared to dCas9-alone parasites targeted to the *NEO* locus. Lastly, the dCas9-Bdf3 EP1-3 clones showed a noticeable time-dependent increase in *EP1/GFP* expression between 48 h and 72 h, whereas in the dCas9-Bdf3 EP1-1 clones, this time-dependent increase was not observed. We conclude that Bdf3 is sufficient to increase transcript levels of *EP1/GFP* when targeted to the *EP1* locus. Given that Bdf3 appears at the *EP1* locus after differentiation from the bloodstream to the procyclic stage is initiated (69), our data support a model in which Bdf3 facilitates transcription at the *EP1* locus once differentiation has been triggered.

### Expression of the dCas9-Bdf3 fusion protein targeted to the *EP1* locus causes a mild growth defect

**Fig 3:**
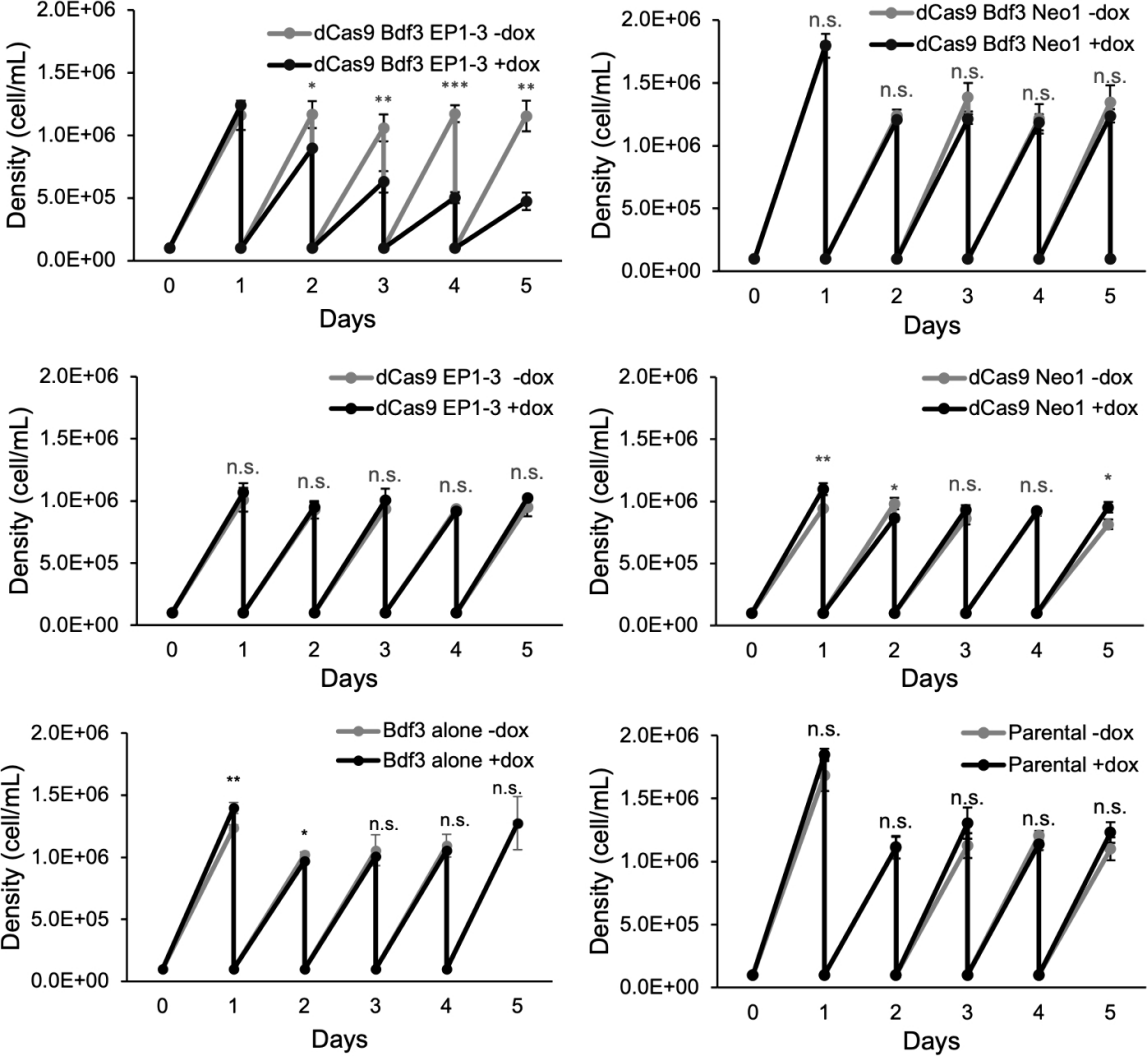
Expression of dCas9-Bdf3 targeted to the *EP1* locus causes a growth defect after induction of dCas9-Bdf3 and the guide RNA. Parasites were seeded at 100,000 cells/mL with or without 1 µg/mL dox and grown for 24 h before being counted and diluted back to 100,000 cells/mL over the course of 5 days. A Student’s *t*-test was used to compare −dox and +dox samples for each parasite strain at each time point. *, *P* < 0.05; **, *P* < 0.01; ***, *P* < 0.001. n.s., not significant.

In order to assess whether expression of *EP1/GFP* induced by dCas9-Bdf3 causes a growth defect, we performed growth assays on both *EP1* and *NEO* guide RNA parasites expressing either the dCas9-Bdf3 fusion protein or dCas9 alone. We observed a mild growth defect in dCas9-Bdf3 parasites targeted to *EP1* after 48 h of induction, but equivalent growth defects were not observed in any other strain we tested (Fig. 3). We do not think the growth defect is caused solely by the expression of the dCas9-Bdf3 fusion protein because parasites induced to express dCas9-Bdf3 targeted to the *NEO* locus do not exhibit a noticeable growth defect.

**Fig 4:**
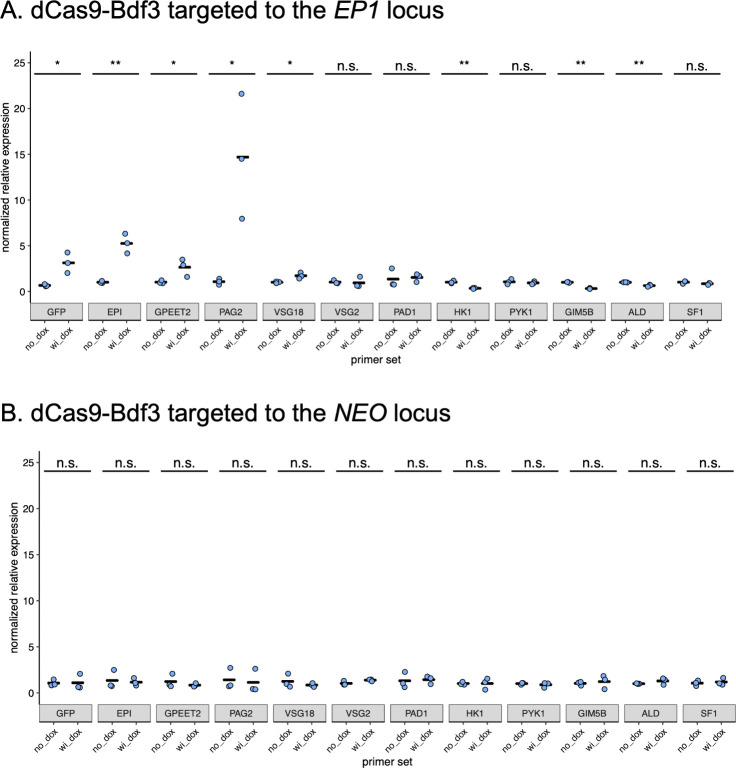
dCas9-Bdf3 targeted to the *EP1* locus results in increased expression of *EP-*related genes. Relative expression of each indicated gene analyzed using quantitative PCR from cDNA generated from RNA extracts of parasites harboring dCas9-Bdf3 targeted to the *EP1* locus (**A**) or dCas9-Bdf3 targeted to the *NEO* locus (**B**). Parasites were treated with dox for 72 h to induce maximal expression of dCas9-Bdf3 and the guide RNA. Scaling was performed such that the average −dox value for each gene was set to 1 to allow for easy comparisons between samples. Note, the active VSG is VSG2. Each dot represents the average of three technical replicates, and the set of three dots for each sample represents three biological replicates for three independent clones. A Student’s *t*-test was performed to compare +dox samples vs −dox samples. *, *P* < 0.05; **, *P* < 0.01; ***, *P* < 0.001, n.s., not significant. wi_dox, with dox; no_dox, no dox.

Multiple researchers have shown that expression of more than one VSG protein can lead to growth defects (17, 84–86), and it is possible that the expression of both *VSG* and *EP1* might cause a similar phenotype. Another possibility relates to the growth-arrested nature of stumpy parasites, which are an intermediate stage that occurs when bloodstream parasites grow to high density; these parasites have been shown to pre-express *EP1* prior to transition to the fly (73). While the monomorph strains we used in this experiment do not form a true stumpy intermediate, we nevertheless tested whether they might have stumpy characteristics by measuring transcript levels of *PAD1* and the active VSG in this parasite strain*—VSG2*. Our expectation was that levels of *PAD1* might increase (87) if parasites were transitioning to a stumpy-like state, while the transcript levels of *VSG2* might decrease in preparation for surface protein remodeling (15). To test this idea, we measured transcript levels of *PAD1* and *VSG2* in parasites induced to express dCas9-Bdf3 targeted to either the *EP1* locus or to the *NEO* locus (Fig. 4). We found no significant difference in transcript levels of either *PAD1* or *VSG2* when parasites expressed dCas9-Bdf3 targeted to the *EP1* locus compared to uninduced parasites. Thus, a transition to a stumpy-like state is likely not driving the observed growth defect in parasites with dCas9-Bdf3 targeted to the *EP1* locus. On the other hand, small molecules that induce expression of *EP1* have been associated with growth defects (72). A study using a procyclin reporter found that some compounds that induced procyclin reporter activity also inhibited the growth of the parasites (88). Thus, while we do not know the exact mechanism for the observed growth defect in dCas9-Bdf3 parasites targeted to the *EP1* locus, other studies have shown that growth inhibition is often associated with an increase in *EP1* transcript levels.

### Transcript levels of *EP1, GPEET, and PAG2* are increased after expression of dCas9-Bdf3 targeted to the *EP1* locus

To verify the flow cytometry results, we measured *EP1* transcript levels directly using qPCR on parasites induced to express dCas9-Bdf3 targeted to the *EP1* locus or to the control *NEO* locus following 3 days of dox treatment. We found that both *EP1* and *GFP* transcript levels were higher in parasites induced to express dCas9-Bdf3 and a guide RNA targeted to *EP1* compared to uninduced parasites (Fig. 4A). Parasites induced to express dCas9-Bdf3 and a guide RNA targeted to the control *NEO* locus did not show an increase in expression of either *EP1* or *GFP*, as expected (Fig. 4B). In addition, expression of a control gene *SF1* (splicing factor 1), which was not expected to change following induction of dCas9-Bdf3, showed no significant change in expression when dCas9-Bdf3 was targeted to either the *EP1* locus or the *NEO* locus (Fig. 4A and B). Because the promoter sequence for *EP1* and *GPEET* are highly similar, we expected that guide RNAs targeted to the *EP1* promoter may also tether dCas9-Bdf3 to the *GPEET* locus, resulting in an increase in *GPEET* expression when both dCas9-Bdf3 and the guide RNA are induced with dox. This hypothesis was supported by an increase in expression of the *GPEET2* gene following induction of both dCas9-Bdf3 and the *EP1* guide RNA (Fig. 4A). No increase in *GPEET2* expression levels was observed in parasites induced to target dCas9-Bdf3 to the *NEO* locus. Given that the *PAG2* gene is found in the same polycistronic unit as *EP1*, we measured the expression levels of *PAG2* following induction of dCas9-Bdf3 targeted to the *EP1* locus. Expression levels of *PAG2* were significantly increased in dCas9-Bdf3 parasites expressing guide RNAs targeted to *EP1* compared to uninduced parasites (Fig. 4A). These changes were not observed when dCas9-Bdf3 was targeted to the control *NEO* locus (Fig. 4B). The observed increase in *EP* and *GPEET* expression in parasites expressing dCas9-Bdf3 targeted to the *EP1* locus is not as large as the changes observed in expression of these genes as parasites are fully differentiating from the bloodstream to the insect stage (72, 89, 90). This observation is consistent with the fact that multiple modes of stabilizing *EP* and *GPEET* transcripts are in play in fully differentiating parasites. Stabilization of *EP* and *GPEET* transcripts has been shown to be mediated through UTR sequences (91–93) and trans-acting factors (94, 95). Environmental factors such as nutrient availability can also influence *GPEET* transcript stability (93, 96). Thus, it is not surprising that transcript levels of *GPEET* and *EP1* observed in dCas9-Bdf3 *EP1*-targeted parasites are not as high as transcript levels observed in differentiating parasites. Finally, multiple different bromodomain protein complexes have been shown to localize to Pol II promoters (38). It is possible that additional transcriptional activators, in addition to Bdf3 and its associated complex members, are required to achieve high transcript levels at the *EP1* promoter or Pol I promoters in general.

Previous work in our lab has shown that treatment of *T. brucei* parasites with either spironolactone or eflornithine causes an increase in expression of *EP1, GFP,* and *PAG2,* and that these changes are accompanied by other changes in transcript levels that occur as parasites transition from the bloodstream to the insect stage (72). These changes include a decrease in expression of genes that are important in the glycolysis pathway, including *PYK1, HK1, ALD*, and *GIM5B*. We were surprised to see that transcript levels of *HK1, GIM5B,* and *ALD* were slightly decreased in parasites where dCas9-Bdf3 was induced and targeted to the *EP1* locus (Fig. 4A). No significant changes in transcript levels for these genes were observed in parasites where dCas9-Bdf3 was induced and targeted to the control *NEO* locus (Fig. 4B). A silent *VSG* gene (VSG18) was also slightly upregulated, which has also been previously observed in conjunction with changes in *EP1* expression (16). The unexpected decrease in transcript levels for genes associated with glycolysis may indicate that a feedback loop of unknown mechanism may exist in parasites for which *EP1* transcript levels are increased. Increases in transcript levels for *EP1* and *GPEET* have been observed in conjunction with a decrease in transcript levels for glycolysis genes in stumpy parasites that undergo transcriptional pre-adaptation to life in the tsetse (73, 89), so there is precedent for these changes occurring together. In addition, knockdown of a hexokinase gene was previously reported to lead to switching from glycosylated EP-procyclin to unglycosylated GPEET-procyclin, supporting the idea that there could be crosstalk between the glycosylation pathway and changes in procyclin expression (96). However, we did not necessarily expect a differentiation program to initiate following an increase in *EP1* transcript levels, and this phenomenon might be worth exploring in future work. It should also be emphasized that the observed difference in transcript levels for glycolysis genes is quite muted compared to the much more dramatic changes in transcript levels observed in bloodstream parasites induced to differentiate to the procyclic form (89, 90).

## DISCUSSION

While the role for bromodomain proteins as transcriptional activators in mammalian cells is well established, whether these proteins are important for facilitating transcription initiation in trypanosomes is less clear. Here, we show that the bromodomain protein Bdf3 is sufficient to increase transcript levels for a gene that is normally silenced in bloodstream parasites (Fig. 2). Both Bdf3 and a guide RNA targeting the *EP1* promoter are required to observe an increase in *EP1* transcript levels (Fig. 2).

One thing that remains unclear is whether acetylation at the promoter of the *EP1* locus is required for Bdf3 to increase transcript levels. In many model systems, bromodomains and histone acetylation domains are often found in one protein, allowing both acetylation of histones and reading of that acetylation to be accomplished by the same protein. Examples include p300, CBP, PCAF, and GCN5, among others (97). In *T. brucei*, none of the seven identified proteins with bromodomains also contain a HAT domain, but there are three other identified *T. brucei* proteins with HAT domains (35). Elegant experiments using immunoprecipitation combined with mass spectrometry have shown that Bdf3 complexes with both Bdf5 and HAT2 (38). HAT2 has been shown to acetylate H4K10 (35, 45), which is found at Pol II promoters (34), and depletion of HAT2 in *T. brucei* alters the site of Pol II transcription initiation in *T. brucei* (45). Bdf3 has also been shown to be important for deposition of H2A.Z at transcription start sites for Pol II promoters (34, 98). However, to our knowledge, aside from Bdf3 (69), it remains unclear whether the histone modifications and proteins observed at Pol II promoters are also found at the Pol I promoters of *EP* and *GPEET* as parasites differentiate from the bloodstream stage to stumpy forms and then to procyclic stages. This is an interesting area for future study. One model is that an increase in histone acetylation at the *EP1* locus during differentiation might facilitate binding of Bdf5, bringing along Bdf3 and HAT2 in the same complex. HAT2 might then acetylate H4K10 and stabilize an interaction between Bdf3 or another bromodomain protein to that residue, facilitating an increase in transcription. An alternate model is that HAT2 might get recruited to the *EP1* locus first by an unknown mechanism. Increases in H4K10ac by HAT2 at the *EP1* promoter may stabilize binding of Bdf3 to H4K10ac and facilitate transcription of *EP1* in differentiating parasites. The targets of the six bromodomain proteins recruited to Pol II transcription start sites in *T. brucei* are not fully characterized, and whether other bromodomain proteins are recruited to Pol I sites during differentiation is unknown. Thus, it is difficult to predict the order of events that drive increased transcription of the *EP1* locus during differentiation. Resynthesis of an H4K10ac-specific antibody and additional antibodies to other acetylated residues, along with time courses to characterize changes in acetylation at the *EP1* locus during differentiation, might provide the tools to start interrogating this question.

To our knowledge, we are the first group to engineer a CRISPR activation-type system in *T. brucei*. It remains unclear whether the tool could be easily adapted to overexpress proteins of interest. Because most Pol II genes are expressed polycistronically from transcription start sites, designing a guide to a specific transcription start site and increasing transcription at that site would be expected to increase transcription of multiple genes simultaneously. In addition, because most transcript levels are regulated post-transcriptionally through 5′ and 3′ UTRs (99, 100), pseudouridination (101), lncRNAs (102), and alternative polyadenylation (103), increasing transcription at the relevant start site may not have any effect on transcript levels for the gene of interest if these other modes of regulation dominate. If a construct containing the gene of interest is placed downstream of a unique promoter and incorporated exogenously, overexpression may be achievable through expression of the dCas9-Bdf3 fusion in conjunction with a guide RNA targeted to the promoter. However, the already existing tet-inducible system (81) may offer the same advantages with less engineering.

One outstanding question that should be addressed in future work is whether Bdf3 is capable of inducing expression from any promoter, or whether there is something unique about the *EP1* locus. This issue has been difficult to address because only a very limited number of genes are transcriptionally regulated in different life cycle stages. In order to rigorously address this question, the promoter in question would ideally be silent in at least one life cycle stage. The promoters upstream of silent *VSG* genes are one example of a region that is silenced in bloodstream parasites, but because the expression site promoters share a great deal of homology, trying to target dCas9-Bdf3 to these promoters may be difficult because the protein would be diluted among many different sites. Another way to address this could be to design a guide RNA to an alternate exogenously introduced promoter. Many *T. brucei* expression constructs utilize either the *GPEET* or the *EP* promoter to drive expression, but utilization of the rRNA promoter is also common. Designing guide RNAs targeting the rRNA promoter and examining the effect on transcript levels of the adjacent gene would address whether Bdf3 is sufficient to increase transcript levels at multiple promoter types. However, because the rRNA promoter is strong and constitutively active in bloodstream parasites, it is possible that the addition of dCas9-Bdf3 would not boost the signal significantly if it is already “maxed out,” so to speak. Thus, a negative result for this experiment may be difficult to interpret. Because *NEO* is controlled by readthrough transcription in our trypanosome system, we did not attempt to control *NEO* expression using guide RNAs. Guide RNAs targeted to the *NEO* gene body were simply used as a sink for dCas9-Bdf3 to avoid untargeted interactions with other regions of the genome, something that is a known issue in the absence of a decoy or irrelevant guide RNA (104, 105). In sum, we are unable to conclude whether Bdf3 acts as a general transcriptional activator or whether the *EP1* locus is uniquely permissive. Additional studies will be required to assess whether Bdf3 can act as a general activator at all Pol I promoters in *T. brucei*. Because we did not test Bdf3’s activity at Pol II promoters, this question must also await additional data from future studies in order to be addressed.

Another useful feature of the engineered dCas9-Bdf3 system is that mutation analyses can now be used to determine what features of the protein are necessary to achieve the increased transcript levels observed when Bdf3 is targeted to the *EP1* locus. Mutation of a conserved tyrosine and asparagine (106, 107) in the bromodomain would allow interrogation for the requirement to bind acetylated histones. Truncations might also assist in determining what portions of the protein are necessary to recruit HAT2 and Bdf5, if any. Finally, Bdf3 is one of six trypanosome bromodomain proteins shown to localize to transcription start sites (16, 34, 38). The dCas9 fusion system that we have engineered can be used to ascertain whether other bromodomain proteins are sufficient to increase transcript levels at the *EP1* locus. The system can also be used to help characterize the many chromatin-associated proteins found in complexes that localize to transcription start sites (38).

## MATERIALS AND METHODS

### Strain details and culture growth

Bloodstream SM *EP1/GFP* parasites (72) were cultured in HMI-9 (108) at 37°C and 5% CO_2_. The dCas9-Bdf3 fusion construct, the dCas9-alone construct, and the Bdf3-alone constructs were transfected into reporter parasites using an AMAXA nucleofector with the X-001 setting. For each transfection, 30–50 million parasites were harvested and resuspended in 100 µL of nucleofector T cell solution with 5–10 µg of NotI-linearized plasmid (New England Biolabs). Parasites were grown for 6 days in culture medium and selected for resistance using 5.0 µg/mL blasticidin. T7 promoter-driven guide RNA constructs were linearized and introduced using the same method except 2.5 µg/mL phleomycin was used for selection. Expression of each introduced protein was confirmed by Western blot (see below).

### Plasmid construction

The plasmid containing the dCas9-Bdf3 fusion sequence was constructed in two halves: the first half consisted of the 3× FLAG tag, La nuclear localization signal (109), and the front half of the dCas9 coding region, and the second half consisted of the back half of the dCas9 coding region, the GS linker, and the Bdf3 coding region. Both halves were synthesized by Twist Biosciences. The front 3× FLAG/La NLS/dCas9 coding region was amplified using forward ggccaagcttatggactacaaggaccac and reverse gcgcgggcccacgtagtacggg primers containing HindIII (New England Biolabs) and ApaI (New England Biolabs) restriction enzyme recognition sites. This section was digested with HindIII and ApaI and cloned into a pLEW100 vector at these same sites. The back dCas9/Glycine-Serine linker/Bdf3 coding region was cut with ApaI and BamHI (New England Biolabs) and cloned into the pLEW100/3× FLAG/La NLS/dCas9 vector at the ApaI and BamHI sites within the vector.

### sgRNA construction

Complementary single-stranded oligos were engineered with BbsI overhangs. Oligos were combined at a concentration of 5 µM each in TE buffer with a reaction volume of 50 µL. The oligos were annealed in a thermocycler as follows: 95°C for 0:01 with a ramp time of 3°C/s, 70°C for 3:00 with a ramp time of 3°C/s, and a hold at 4°C with a ramp time of 0.1°C/s. pT7sgRNA (78) was a gift from David Horn (Addgene plasmid # 111820; http://n2t.net/addgene: 111820; RRID: Addgene_111820). The pT7sgRNA vector was linearized with BbsI-HF (New England Biolabs) and purified. Linearized pT7sgRNA (150 ng) was combined with annealed oligo at a concentration of 2 µM and ligated together using Quick Ligase (New England Biolabs) in a 20 µL reaction for 5 min at 25°C. Ligated plasmids were transformed into electrocompetent cells to screen for correct integration of the oligos. The oligo sequences used were as follows: EP1-1 forward, agggtgggcgtgcattgaaaatag; EP1-1 reverse, aaacctattttcaatgcacgccca; EP1-3 forward, aggggctgttccgtgtctctgggt; EP1-3 reverse, aaacacccagagacacggaacagc; Neo1 forward, aggggatctggacgaagagcatca; Neo1 reverse, aaactgatgctcttcgtccagatc.

### Western blotting

SM *EP1/GFP* dCas9-Bdf3, dCas9-alone, or Bdf3-alone parasites were treated with or without 1 µg/mL dox, and 8 million parasites were harvested after 24 h or 48 h of treatment. Harvested parasites were resuspended in 2× Laemmli buffer and boiled at 95°C for 10 min. The samples were loaded onto a 4-20% gradient polyacrylamide gel, and protein was transferred to the PVDF membrane. The membrane was blocked in 7 mL of 5% milk in Tris-buffered saline (TBS), then incubated overnight at 4°C in M2 mouse anti-FLAG (Sigma), rabbit anti-FLAG (Cell Signaling), or the control anti-tubulin (a kind gift from George Cross) in 5% milk in TBS at a 1:1,000 dilution. After three washes in TBS with 0.1% Tween-20, the membrane was incubated at room temperature for 1 h in goat anti-mouse or goat anti-rabbit fluorescently labeled antibody (Bio-Rad) at a 1:10,000 dilution in 5% milk in TBS. After all antibody incubations, the membrane was washed three times in TBS with 0.1% Tween-20 and a final wash in TBS alone. The membrane was imaged on a LI-COR Odyssey instrument.

### Flow cytometry

SM *EP1/GFP* dCas9-Bdf3 or dCas9 cells were prepared at a density of 15,000 cells/mL and treated with or without 1 μg/mL dox in 2 mL of culture medium. Parasites were diluted in fresh media with dox as needed to maintain them under 1 million/mL. After 2 and 3 days of growth, 1 mL of culture was harvested and analyzed by flow cytometry for *EP1/GFP* expression. All samples were analyzed with a Novocyte 2000R from ACEA Biosciences.

### Analysis of parasite growth

Parasites were prepared at a density of 100,000 cells/mL and grown in 96-well plates with a starting volume of 200 µL HMI-9 medium for 24 h with or without 1 µg/mL dox. After 24 h of growth, 20 µL of culture was analyzed by flow cytometry, and the number of parasites in the live gate was calculated. Cultures were diluted back down to 100,000 cells/mL using the number of cells determined by the live gate. This procedure occurred daily for a total of 5 days.

### Quantitative PCR

RNA was isolated from parasites using the Direct-zol RNA MiniPrep kit from Genesee, which includes DNase treatment. cDNA was generated using the SuperScript IV VILO Master Mix (Fisher Scientific) according to the manufacturer’s instructions starting with 1 µg of RNA. cDNA was amplified with primers combined with 2× SYBR Green master mix (Life Technologies) and analyzed on an Eppendorf Realplex2 instrument. Primers used were as follows: Tb427.10.10260 EP1, tctgctcgctattcttctgttc, cctttgcctcccttagtaagac; Tb927.6.510 GPEET agtcggctagcaacgttatc, ttctggtccggtctcttct; Tb927.9.11600, GIM5B, ttgcgaggatgggtgatg, gggtttggagagggaagttaat; Tb927.10.2010, HK1, gtcagcacttactcccatcaa, acgacgcatcgtcaatatcc; Tb927.10.5620, ALD, gtctgaagctgttgttcgtttc, cacctcaggctccacaatag; Tb927.10.10220, PAG2, aggagatacgaggaatgagaca, tcttcaaacgcccggtaag; Tb927.10.14140 PYK1, gagaaggttggcacaaagga, tcacaccgtcgtcaacataaa, GFP, ctacaacagccacaaggtctat, ggtgttctgctggtagtg; Tb927.10.9400, SF1, ggtatggttcatcaggagttgg, cgttagcactggtatccttcag.

## SUPPLEMENTAL MATERIAL

Supplemental material msphere.00021-26-s0001.docx Figure S1 and supplemental table legends.

Table S1 msphere.00021-26-s0002.txt *P* values from ANOVA and Tukey HSD *post-hoc* analysis for 48-h induction of dCas9 and guide RNAs.

Table S2 msphere.00021-26-s0003.txt *P* values from ANOVA and Tukey HSD *post-hoc* analysis for 72-h induction of dCas9 and guide RNAs.

ASM does not own the copyrights to Supplemental Material that may be linked to, or accessed through, an article. The authors have granted ASM a non-exclusive, world-wide license to publish the Supplemental Material files. Please contact the corresponding author directly for reuse.

## References

[1] Alsan M. 2015. The effect of the TseTse fly on African development. Am Econ Rev 105:382–410. doi:10.1257/aer.20130604

[2] Shaw APM, Cecchi G, Wint GRW, Mattioli RC, Robinson TP. 2014. Mapping the economic benefits to livestock keepers from intervening against bovine trypanosomosis in Eastern Africa. Prev Vet Med 113:197–210. doi:10.1016/j.prevetmed.2013.10.024 24275205

[3] Cross GAM, Kim H-S, Wickstead B. 2014. Capturing the variant surface glycoprotein repertoire (the VSGnome) of Trypanosoma brucei Lister 427. Mol Biochem Parasitol 195:59–73. doi:10.1016/j.molbiopara.2014.06.004 24992042

[4] Hellemond JJ van, Bakker BM, Tielens AGM. 2005. Energy metabolism and its compartmentation in Trypanosoma brucei. Adv Microb Physiol 50:199–226. doi:10.1016/S0065-2911(05)50005-5 16221581

[5] van Grinsven KWA, Van Den Abbeele J, Van den Bossche P, van Hellemond JJ, Tielens AGM. 2009. Adaptations in the glucose metabolism of procyclic Trypanosoma brucei isolates from tsetse flies and during differentiation of bloodstream forms. Eukaryot Cell 8:1307–1311. doi:10.1128/EC.00091-09 19542311 PMC2725551

[6] Flynn IW, Bowman IBR. 1973. The metabolism of carbohydrate by pleomorphic African trypanosomes. Comp Biochem Physiol B Comp Biochem 45:25–42. doi:10.1016/0305-0491(73)90281-2 4719992

[7] Hannaert V, Bringaud F, Opperdoes FR, Michels PA. 2003. Evolution of energy metabolism and its compartmentation in Kinetoplastida. Kinetoplastid Biol Dis 2:11. doi:10.1186/1475-9292-2-11 14613499 PMC317351

[8] Roditi I, Carrington M, Turner M. 1987. Expression of a polypeptide containing a dipeptide repeat is confined to the insect stage of Trypanosoma brucei. Nature 325:272–274. doi:10.1038/325272a0 3808022

[9] Roditi I, Schwarz H, Pearson TW, Beecroft RP, Liu MK, Richardson JP, Bühring HJ, Pleiss J, Bülow R, Williams RO. 1989. Procyclin gene expression and loss of the variant surface glycoprotein during differentiation of Trypanosoma brucei. J Cell Biol 108:737–746. doi:10.1083/jcb.108.2.737 2645304 PMC2115453

[10] Vassella E, Acosta-Serrano A, Studer E, Lee SH, Englund PT, Roditi I. 2001. Multiple procyclin isoforms are expressed differentially during the development of insect forms of Trypanosoma brucei. J Mol Biol 312:597–607. doi:10.1006/jmbi.2001.5004 11575917

[11] Acosta-Serrano A, Vassella E, Liniger M, Kunz Renggli C, Brun R, Roditi I, Englund PT. 2001. The surface coat of procyclic Trypanosoma brucei: programmed expression and proteolytic cleavage of procyclin in the tsetse fly. Proc Natl Acad Sci USA 98:1513–1518. doi:10.1073/pnas.98.4.1513 11171982 PMC29288

[12] Vassella E, Oberle M, Urwyler S, Renggli CK, Studer E, Hemphill A, Fragoso C, Bütikofer P, Brun R, Roditi I. 2009. Major surface glycoproteins of insect forms of Trypanosoma brucei are not essential for cyclical transmission by tsetse. PLoS One 4:e4493. doi:10.1371/journal.pone.0004493 19223969 PMC2637416

[13] Sheader K, Vaughan S, Minchin J, Hughes K, Gull K, Rudenko G. 2005. Variant surface glycoprotein RNA interference triggers a precytokinesis cell cycle arrest in African trypanosomes. Proc Natl Acad Sci USA 102:8716–8721. doi:10.1073/pnas.0501886102 15937117 PMC1150830

[14] Nagamune K, Nozaki T, Maeda Y, Ohishi K, Fukuma T, Hara T, Schwarz RT, Sutterlin C, Brun R, Riezman H, Kinoshita T. 2000. Critical roles of glycosylphosphatidylinositol for Trypanosoma brucei. Proc Natl Acad Sci USA 97:10336–10341. doi:10.1073/pnas.180230697 10954751 PMC27025

[15] Amiguet-Vercher A, Pérez-Morga D, Pays A, Poelvoorde P, Van Xong H, Tebabi P, Vanhamme L, Pays E. 2004. Loss of the mono-allelic control of the VSG expression sites during the development of Trypanosoma brucei in the bloodstream. Mol Microbiol 51:1577–1588. doi:10.1111/j.1365-2958.2003.03937.x 15009886

[16] Schulz D, Mugnier MR, Paulsen E-M, Kim H-S, Chung CW, Tough DF, Rioja I, Prinjha RK, Papavasiliou FN, Debler EW. 2015. Bromodomain proteins contribute to maintenance of bloodstream form stage identity in the African trypanosome. PLoS Biol 13:e1002316. doi:10.1371/journal.pbio.1002316 26646171 PMC4672894

[17] Batram C, Jones NG, Janzen CJ, Markert SM, Engstler M. 2014. Expression site attenuation mechanistically links antigenic variation and development in Trypanosoma brucei. eLife 3:e02324. doi:10.7554/eLife.02324 24844706 PMC4027811

[18] Zimmermann H, Subota I, Batram C, Kramer S, Janzen CJ, Jones NG, Engstler M. 2017. A quorum sensing-independent path to stumpy development in Trypanosoma brucei. PLoS Pathog 13:e1006324. doi:10.1371/journal.ppat.1006324 28394929 PMC5398725

[19] Clayton CE, Fueri JP, Itzhaki JE, Bellofatto V, Sherman DR, Wisdom GS, Vijayasarathy S, Mowatt MR. 1990. Transcription of the procyclic acidic repetitive protein genes of Trypanosoma brucei. Mol Cell Biol 10:3036–3047. doi:10.1128/mcb.10.6.3036-3047.1990 2342468 PMC360668

[20] Biebinger S, Rettenmaier S, Flaspohler J, Hartmann C, Peña-Diaz J, Wirtz LE, Hotz HR, Barry JD, Clayton C. 1996. The PARP promoter of Trypanosoma brucei is developmentally regulated in a chromosomal context. Nucleic Acids Res 24:1202–1211. doi:10.1093/nar/24.7.1202 8614620 PMC145797

[21] Brown SD, Huang J, Van der Ploeg LH. 1992. The promoter for the procyclic acidic repetitive protein (PARP) genes of Trypanosoma brucei shares features with RNA polymerase I promoters. Mol Cell Biol 12:2644–2652. doi:10.1128/mcb.12.6.2644-2652.1992 1588962 PMC364458

[22] Günzl A, Bruderer T, Laufer G, Schimanski B, Tu L-C, Chung H-M, Lee P-T, Lee MG-S. 2003. RNA polymerase I transcribes procyclin genes and variant surface glycoprotein gene expression sites in Trypanosoma brucei. Eukaryot Cell 2:542–551. doi:10.1128/EC.2.3.542-551.2003 12796299 PMC161450

[23] Rudenko G, Lee MG, Van der Ploeg LH. 1992. The PARP and VSG genes of Trypanosoma brucei do not resemble RNA polymerase II transcription units in sensitivity to Sarkosyl in nuclear run-on assays. Nucleic Acids Res 20:303–306. doi:10.1093/nar/20.2.303 1371345 PMC310370

[24] Berriman M, Ghedin E, Hertz-Fowler C, Blandin G, Renauld H, Bartholomeu DC, Lennard NJ, Caler E, Hamlin NE, Haas B, et al.. 2005. The genome of the African trypanosome Trypanosoma brucei. Science 309:416–422. doi:10.1126/science.1112642 16020726

[25] Clayton CE. 2016. Gene expression in Kinetoplastids. Curr Opin Microbiol 32:46–51. doi:10.1016/j.mib.2016.04.018 27177350

[26] Campbell DA, Sturm NR, Yu MC. 2000. Transcription of the kinetoplastid spliced leader RNA gene. Parasitol Today 16:78–82. doi:10.1016/s0169-4758(99)01545-8 10652494

[27] Gilinger G, Bellofatto V. 2001. Trypanosome spliced leader RNA genes contain the first identified RNA polymerase II gene promoter in these organisms. Nucleic Acids Res 29:1556–1564. doi:10.1093/nar/29.7.1556 11266558 PMC31286

[28] Budzak J, Jones R, Tschudi C, Kolev NG, Rudenko G. 2022. An assembly of nuclear bodies associates with the active VSG expression site in African trypanosomes. Nat Commun 13:101. doi:10.1038/s41467-021-27625-6 35013170 PMC8748868

[29] Faria J, Luzak V, Müller LSM, Brink BG, Hutchinson S, Glover L, Horn D, Siegel TN. 2021. Spatial integration of transcription and splicing in a dedicated compartment sustains monogenic antigen expression in African trypanosomes. Nat Microbiol 6:289–300. doi:10.1038/s41564-020-00833-4 33432154 PMC7610597

[30] Cordon-Obras C, Gomez-Liñan C, Torres-Rusillo S, Vidal-Cobo I, Lopez-Farfan D, Barroso-Del Jesus A, Rojas-Barros D, Carrington M, Navarro M. 2022. Identification of sequence-specific promoters driving polycistronic transcription initiation by RNA polymerase II in trypanosomes. Cell Rep 38:110221. doi:10.1016/j.celrep.2021.110221 35021094

[31] Luzak V, Osses E, Danese A, Odendaal C, Cosentino RO, Stricker SH, Haanstra JR, Erhard F, Siegel TN. 2025. SLAM-seq reveals independent contributions of RNA processing and stability to gene expression in African trypanosomes. Nucleic Acids Res 53:gkae1203. doi:10.1093/nar/gkae1203 39673807 PMC11797058

[32] Jenuwein T, Allis CD. 2001. Translating the histone code. Science 293:1074–1080. doi:10.1126/science.1063127 11498575

[33] Maree JP, Tvardovskiy A, Ravnsborg T, Jensen ON, Rudenko G, Patterton H-G. 2022. Trypanosoma brucei histones are heavily modified with combinatorial post-translational modifications and mark Pol II transcription start regions with hyperacetylated H2A. Nucleic Acids Res 50:9705–9723. doi:10.1093/nar/gkac759 36095123 PMC9508842

[34] Siegel TN, Hekstra DR, Kemp LE, Figueiredo LM, Lowell JE, Fenyo D, Wang X, Dewell S, Cross GAM. 2009. Four histone variants mark the boundaries of polycistronic transcription units in Trypanosoma brucei. Genes Dev 23:1063–1076. doi:10.1101/gad.1790409 19369410 PMC2682952

[35] Kawahara T, Siegel TN, Ingram AK, Alsford S, Cross GAM, Horn D. 2008. Two essential MYST-family proteins display distinct roles in histone H4K10 acetylation and telomeric silencing in trypanosomes. Mol Microbiol 69:1054–1068. doi:10.1111/j.1365-2958.2008.06346.x 18631159 PMC2556858

[36] Siegel TN, Kawahara T, Degrasse JA, Janzen CJ, Horn D, Cross GAM. 2008. Acetylation of histone H4K4 is cell cycle regulated and mediated by HAT3 in Trypanosoma brucei. Mol Microbiol 67:762–771. doi:10.1111/j.1365-2958.2007.06079.x 18179414 PMC2253726

[37] Wang Q-P, Kawahara T, Horn D. 2010. Histone deacetylases play distinct roles in telomeric VSG expression site silencing in African trypanosomes. Mol Microbiol 77:1237–1245. doi:10.1111/j.1365-2958.2010.07284.x 20624217 PMC2941730

[38] Staneva DP, Carloni R, Auchynnikava T, Tong P, Rappsilber J, Jeyaprakash AA, Matthews KR, Allshire RC. 2021. A systematic analysis of Trypanosoma brucei chromatin factors identifies novel protein interaction networks associated with sites of transcription initiation and termination. Genome Res 31:2138–2154. doi:10.1101/gr.275368.121 34407985 PMC8559703

[39] Frederiks F, van Welsem T, Oudgenoeg G, Heck AJR, Janzen CJ, van Leeuwen F. 2010. Heterologous expression reveals distinct enzymatic activities of two DOT1 histone methyltransferases of Trypanosoma brucei. J Cell Sci 123:4019–4023. doi:10.1242/jcs.073882 21084562

[40] Figueiredo LM, Janzen CJ, Cross GAM. 2008. A histone methyltransferase modulates antigenic variation in African trypanosomes. PLoS Biol 6:e161. doi:10.1371/journal.pbio.0060161 18597556 PMC2443197

[41] Lowell JE, Kaiser F, Janzen CJ, Cross GAM. 2005. Histone H2AZ dimerizes with a novel variant H2B and is enriched at repetitive DNA in Trypanosoma brucei. J Cell Sci 118:5721–5730. doi:10.1242/jcs.02688 16303849

[42] Wedel C, Förstner KU, Derr R, Siegel TN. 2017. GT‐rich promoters can drive RNA pol II transcription and deposition of H2A.Z in African trypanosomes. EMBO J 36:2581–2594. doi:10.15252/embj.201695323 28701485 PMC5579346

[43] Wright JR, Siegel TN, Cross GAM. 2010. Histone H3 trimethylated at lysine 4 is enriched at probable transcription start sites in Trypanosoma brucei. Mol Biochem Parasitol 172:141–144. doi:10.1016/j.molbiopara.2010.03.013 20347883 PMC2875994

[44] ElBashir R, Vanselow JT, Kraus A, Janzen CJ, Siegel TN, Schlosser A. 2015. Fragment ion patchwork quantification for measuring site-specific acetylation degrees. Anal Chem 87:9939–9945. doi:10.1021/acs.analchem.5b02517 26335048

[45] Kraus AJ, Vanselow JT, Lamer S, Brink BG, Schlosser A, Siegel TN. 2020. Distinct roles for H4 and H2A.Z acetylation in RNA transcription in African trypanosomes. Nat Commun 11:1498. doi:10.1038/s41467-020-15274-0 32198348 PMC7083915

[46] Hughes K, Wand M, Foulston L, Young R, Harley K, Terry S, Ersfeld K, Rudenko G. 2007. A novel ISWI is involved in VSG expression site downregulation in African trypanosomes. EMBO J 26:2400–2410. doi:10.1038/sj.emboj.7601678 17431399 PMC1864976

[47] Figueiredo LM, Cross GAM. 2010. Nucleosomes are depleted at the VSG expression site transcribed by RNA polymerase I in African trypanosomes. Eukaryot Cell 9:148–154. doi:10.1128/EC.00282-09 19915072 PMC2805297

[48] Stanne TM, Rudenko G. 2010. Active VSG expression sites in Trypanosoma brucei are depleted of nucleosomes. Eukaryot Cell 9:136–147. doi:10.1128/EC.00281-09 19915073 PMC2805301

[49] Glover L, Horn D. 2014. Locus-specific control of DNA resection and suppression of subtelomeric VSG recombination by HAT3 in the African trypanosome. Nucleic Acids Res 42:12600–12613. doi:10.1093/nar/gku900 25300492 PMC4227765

[50] Tamkun JW, Deuring R, Scott MP, Kissinger M, Pattatucci AM, Kaufman TC, Kennison JA. 1992. brahma: a regulator of Drosophila homeotic genes structurally related to the yeast transcriptional activator SNF2SWI2. Cell 68:561–572. doi:10.1016/0092-8674(92)90191-e 1346755

[51] Haynes SR, Dollard C, Winston F, Beck S, Trowsdale J, Dawid IB. 1992. The bromodomain: a conserved sequence found in human, Drosophila and yeast proteins. Nucleic Acids Res 20:2603–2603. doi:10.1093/nar/20.10.2603 1350857 PMC312404

[52] Brownell JE, Zhou J, Ranalli T, Kobayashi R, Edmondson DG, Roth SY, Allis CD. 1996. Tetrahymena histone acetyltransferase A: a homolog to yeast Gcn5p linking histone acetylation to gene activation. Cell 84:843–851. doi:10.1016/S0092-8674(00)81063-6 8601308

[53] Ogryzko VV, Schiltz RL, Russanova V, Howard BH, Nakatani Y. 1996. The transcriptional coactivators p300 and CBP are histone acetyltransferases. Cell 87:953–959. doi:10.1016/S0092-8674(00)82001-2 8945521

[54] Bannister AJ, Kouzarides T. 1996. The CBP co-activator is a histone acetyltransferase. Nature 384:641–643. doi:10.1038/384641a0 8967953

[55] Di Micco R, Fontanals-Cirera B, Low V, Ntziachristos P, Yuen SK, Lovell CD, Dolgalev I, Yonekubo Y, Zhang G, Rusinova E, Gerona-Navarro G, Cañamero M, Ohlmeyer M, Aifantis I, Zhou M-M, Tsirigos A, Hernando E. 2014. Control of embryonic stem cell identity by BRD4-dependent transcriptional elongation of super-enhancer-associated pluripotency genes. Cell Rep 9:234–247. doi:10.1016/j.celrep.2014.08.055 25263550 PMC4317728

[56] Rodriguez RM, Suarez-Alvarez B, Salvanés R, Huidobro C, Toraño EG, Garcia-Perez JL, Lopez-Larrea C, Fernandez AF, Bueno C, Menendez P, Fraga MF. 2014. Role of BRD4 in hematopoietic differentiation of embryonic stem cells. Epigenetics 9:566–578. doi:10.4161/epi.27711 24445267 PMC4121367

[57] Horne GA, Stewart HJS, Dickson J, Knapp S, Ramsahoye B, Chevassut T. 2015. Nanog requires BRD4 to maintain murine embryonic stem cell pluripotency and is suppressed by bromodomain inhibitor JQ1 together with Lefty1. Stem Cells Dev 24:879–891. doi:10.1089/scd.2014.0302 25393219 PMC4367495

[58] Fleck K, McNutt S, Chu F, Jeffers V. 2023. An apicomplexan bromodomain protein, TgBDP1, associates with diverse epigenetic factors to regulate essential transcriptional processes in Toxoplasma gondii. mBio 14:e03573-22. doi:10.1128/mbio.03573-22 37350586 PMC10470533

[59] Jones NG, Geoghegan V, Moore G, Carnielli JBT, Newling K, Calderón F, Gabarró R, Martín J, Prinjha RK, Rioja I, Wilkinson AJ, Mottram JC. 2022. Bromodomain factor 5 is an essential regulator of transcription in Leishmania. Nat Commun 13:4071. doi:10.1038/s41467-022-31742-1 35831302 PMC9279504

[60] Alonso VL, Villanova GV, Ritagliati C, Machado Motta MC, Cribb P, Serra EC. 2014. Trypanosoma cruzi bromodomain factor 3 binds acetylated α-tubulin and concentrates in the flagellum during metacyclogenesis. Eukaryot Cell 13:822–831. doi:10.1128/EC.00341-13 24747213 PMC4054268

[61] Alonso VL, Ritagliati C, Cribb P, Cricco JA, Serra EC. 2016. Overexpression of bromodomain factor 3 in Trypanosoma cruzi (TcBDF3) affects differentiation of the parasite and protects it against bromodomain inhibitors. FEBS J 283:2051–2066. doi:10.1111/febs.13719 27007774

[62] Tang J, Yeoh LM, Grotz MD, Goodman CD, Chisholm SA, Nguyen HHT, Yu C, Pareek K, McPherson F, Cozijnsen A, Hustadt SA, Josling GA, Day KP, Schulz D, McFadden GI, de Koning-Ward TF, Petter M, Duffy MF. 2025. PfGCN5 is essential for Plasmodium falciparum survival and transmission and regulates Pf H2B.Z acetylation and chromatin structure. Nucleic Acids Res 53:gkaf218. doi:10.1093/nar/gkaf218 40156869 PMC11954527

[63] Miao J, Wang C, Lucky AB, Liang X, Min H, Adapa SR, Jiang R, Kim K, Cui L. 2021. A unique GCN5 histone acetyltransferase complex controls erythrocyte invasion and virulence in the malaria parasite Plasmodium falciparum. PLoS Pathog 17:e1009351. doi:10.1371/journal.ppat.1009351 34403450 PMC8396726

[64] Tian J, Dai B, Gong L, Wang P, Ding H, Xia S, Sun W, Ren C, Shen J, Liu M. 2022. JQ-1 ameliorates schistosomiasis liver granuloma in mice by suppressing male and female reproductive systems and egg development of Schistosoma japonicum. PLoS Negl Trop Dis 16:e0010661. doi:10.1371/journal.pntd.0010661 35943970 PMC9362908

[65] Ashby EC, Havens JL, Rollosson LM, Hardin J, Schulz D. 2023. Chemical inhibition of bromodomain proteins in insect-stage African trypanosomes perturbs silencing of the variant surface glycoprotein repertoire and results in widespread changes in the transcriptome. Microbiol Spectr 11:e00147-23. doi:10.1128/spectrum.00147-23 37097159 PMC10269879

[66] Figueiredo LM, Cross GAM, Janzen CJ. 2009. Epigenetic regulation in African trypanosomes: a new kid on the block. Nat Rev Microbiol 7:504–513. doi:10.1038/nrmicro2149 19528957

[67] Miller G, Rollosson LM, Saada C, Wade SJ, Schulz D. 2023. Adaptation of CUT&RUN for use in African trypanosomes. PLoS One 18:e0292784. doi:10.1371/journal.pone.0292784 37988382 PMC10662711

[68] Ziegelbauer K, Quinten M, Schwarz H, Pearson TW, Overath P. 1990. Synchronous differentiation of Trypanosoma brucei from bloodstream to procyclic forms in vitro. Eur J Biochem 192:373–378. doi:10.1111/j.1432-1033.1990.tb19237.x 1698624

[69] Ashby E, Paddock L, Betts HL, Liao J, Miller G, Porter A, Rollosson LM, Saada C, Tang E, Wade SJ, Hardin J, Schulz D. 2022. Genomic occupancy of the bromodomain protein Bdf3 is dynamic during differentiation of African trypanosomes from bloodstream to procyclic forms. mSphere 7:e00023-22. doi:10.1128/msphere.00023-22 35642518 PMC9241505

[70] Gilbert LA, Larson MH, Morsut L, Liu Z, Brar GA, Torres SE, Stern-Ginossar N, Brandman O, Whitehead EH, Doudna JA, Lim WA, Weissman JS, Qi LS. 2013. CRISPR-mediated modular RNA-guided regulation of transcription in eukaryotes. Cell 154:442–451. doi:10.1016/j.cell.2013.06.044 23849981 PMC3770145

[71] Gilbert LA, Horlbeck MA, Adamson B, Villalta JE, Chen Y, Whitehead EH, Guimaraes C, Panning B, Ploegh HL, Bassik MC, Qi LS, Kampmann M, Weissman JS. 2014. Genome-scale CRISPR-mediated control of gene repression and activation. Cell 159:647–661. doi:10.1016/j.cell.2014.09.029 25307932 PMC4253859

[72] Walsh ME, Naudzius EM, Diaz SJ, Wismar TW, Martchenko Shilman M, Schulz D. 2020. Identification of clinically approved small molecules that inhibit growth and affect transcript levels of developmentally regulated genes in the African trypanosome. PLoS Negl Trop Dis 14:e0007790. doi:10.1371/journal.pntd.0007790 32168320 PMC7094864

[73] Rico E, Rojas F, Mony BM, Szoor B, Macgregor P, Matthews KR. 2013. Bloodstream form pre-adaptation to the tsetse fly in Trypanosoma brucei. Front Cell Infect Microbiol 3:78. doi:10.3389/fcimb.2013.00078 24294594 PMC3827541

[74] Chen X, Zaro JL, Shen W-C. 2013. Fusion protein linkers: property, design and functionality. Adv Drug Deliv Rev 65:1357–1369. doi:10.1016/j.addr.2012.09.039 23026637 PMC3726540

[75] Qi LS, Larson MH, Gilbert LA, Doudna JA, Weissman JS, Arkin AP, Lim WA. 2013. Repurposing CRISPR as an RNA-guided platform for sequence-specific control of gene expression. Cell 152:1173–1183. doi:10.1016/j.cell.2013.02.022 23452860 PMC3664290

[76] Pfeiffer ML, Winkler J, Van Damme D, Jacobs TB, Nowack MK. 2022. Conditional and tissue-specific approaches to dissect essential mechanisms in plant development. Curr Opin Plant Biol 65:102119. doi:10.1016/j.pbi.2021.102119 34653951 PMC7612331

[77] Xiao B, Yin S, Hu Y, Sun M, Wei J, Huang Z, Wen Y, Dai X, Chen H, Mu J, Cui L, Jiang L. 2019. Epigenetic editing by CRISPR/dCas9 in Plasmodium falciparum. Proc Natl Acad Sci USA 116:255–260. doi:10.1073/pnas.1813542116 30584102 PMC6320497

[78] Rico E, Jeacock L, Kovářová J, Horn D. 2018. Inducible high-efficiency CRISPR-Cas9-targeted gene editing and precision base editing in African trypanosomes. Sci Rep 8:7960. doi:10.1038/s41598-018-26303-w 29785042 PMC5962531

[79] Shaw S, Knüsel S, Hoenner S, Roditi I. 2020. A transient CRISPR/Cas9 expression system for genome editing in Trypanosoma brucei. BMC Res Notes 13:268. doi:10.1186/s13104-020-05089-z 32493474 PMC7268226

[80] Beneke T, Madden R, Makin L, Valli J, Sunter J, Gluenz E. 2017. A CRISPR Cas9 high-throughput genome editing toolkit for kinetoplastids. R Soc Open Sci 4:170095. doi:10.1098/rsos.170095 28573017 PMC5451818

[81] Wirtz E, Leal S, Ochatt C, Cross GA. 1999. A tightly regulated inducible expression system for conditional gene knock-outs and dominant-negative genetics in Trypanosoma brucei. Mol Biochem Parasitol 99:89–101. doi:10.1016/s0166-6851(99)00002-x 10215027

[82] Huang J, Van der Ploeg LH. 1991. Requirement of a polypyrimidine tract for trans‐splicing in trypanosomes: discriminating the PARP promoter from the immediately adjacent 3′ splice acceptor site. EMBO J 10:3877–3885. doi:10.1002/j.1460-2075.1991.tb04957.x 1935907 PMC453125

[83] Peng D, Tarleton R. 2015. EuPaGDT: a web tool tailored to design CRISPR guide RNAs for eukaryotic pathogens. Microb Genom 1:e000033. doi:10.1099/mgen.0.000033 28348817 PMC5320623

[84] Pena AC, Pimentel MR, Manso H, Vaz-Drago R, Pinto-Neves D, Aresta-Branco F, Rijo-Ferreira F, Guegan F, Pedro Coelho L, Carmo-Fonseca M, Barbosa-Morais NL, Figueiredo LM. 2014. Trypanosoma brucei histone H1 inhibits RNA polymerase I transcription and is important for parasite fitness in vivo. Mol Microbiol 93:645–663. doi:10.1111/mmi.12677 24946224 PMC4285223

[85] Glover L, Hutchinson S, Alsford S, Horn D. 2016. VEX1 controls the allelic exclusion required for antigenic variation in trypanosomes. Proc Natl Acad Sci USA 113:7225–7230. doi:10.1073/pnas.1600344113 27226299 PMC4932947

[86] Aresta-Branco F, Sanches-Vaz M, Bento F, Rodrigues JA, Figueiredo LM. 2019. African trypanosomes expressing multiple VSGs are rapidly eliminated by the host immune system. Proc Natl Acad Sci USA 116:20725–20735. doi:10.1073/pnas.1905120116 31554700 PMC6789922

[87] Dean S, Marchetti R, Kirk K, Matthews KR. 2009. A surface transporter family conveys the trypanosome differentiation signal. Nature 459:213–217. doi:10.1038/nature07997 19444208 PMC2685892

[88] Wenzler T, Schumann Burkard G, Schmidt RS, Mäser P, Bergner A, Roditi I, Brun R. 2016. A new approach to chemotherapy: drug-induced differentiation kills African trypanosomes. Sci Rep 6:22451. doi:10.1038/srep22451 26931380 PMC4773815

[89] Kabani S, Fenn K, Ross A, Ivens A, Smith TK, Ghazal P, Matthews K. 2009. Genome-wide expression profiling of in vivo-derived bloodstream parasite stages and dynamic analysis of mRNA alterations during synchronous differentiation in Trypanosoma brucei. BMC Genomics 10:427. doi:10.1186/1471-2164-10-427 19747379 PMC2753553

[90] Queiroz R, Benz C, Fellenberg K, Hoheisel JD, Clayton C. 2009. Transcriptome analysis of differentiating trypanosomes reveals the existence of multiple post-transcriptional regulons. BMC Genomics 10:495. doi:10.1186/1471-2164-10-495 19857263 PMC2772864

[91] Furger A, Schürch N, Kurath U, Roditi I. 1997. Elements in the 3′ untranslated region of procyclin mRNA regulate expression in insect forms of Trypanosoma brucei by modulating RNA stability and translation. Mol Cell Biol 17:4372–4380. doi:10.1128/MCB.17.8.4372 9234695 PMC232291

[92] Hehl A, Vassella E, Braun R, Roditi I. 1994. A conserved stem-loop structure in the 3’ untranslated region of procyclin mRNAs regulates expression in Trypanosoma brucei. Proc Natl Acad Sci USA 91:370–374. doi:10.1073/pnas.91.1.370 8278396 PMC42949

[93] Vassella E, Den Abbeele JV, Bütikofer P, Renggli CK, Furger A, Brun R, Roditi I. 2000. A major surface glycoprotein of Trypanosoma brucei is expressed transiently during development and can be regulated post-transcriptionally by glycerol or hypoxia. Genes Dev 14:615–626. doi:10.1101/gad.14.5.615 10716949 PMC316419

[94] Walrad P, Paterou A, Acosta-Serrano A, Matthews KR. 2009. Differential trypanosome surface coat regulation by a CCCH protein that co-associates with procyclin mRNA cis-elements. PLoS Pathog 5:e1000317. doi:10.1371/journal.ppat.1000317 19247446 PMC2642730

[95] Walrad PB, Capewell P, Fenn K, Matthews KR. 2012. The post-transcriptional trans-acting regulator, TbZFP3, co-ordinates transmission-stage enriched mRNAs in Trypanosoma brucei. Nucleic Acids Res 40:2869–2883. doi:10.1093/nar/gkr1106 22140102 PMC3326296

[96] Morris JC, Wang Z, Drew ME, Englund PT. 2002. Glycolysis modulates trypanosome glycoprotein expression as revealed by an RNAi library. EMBO J 21:4429–4438. doi:10.1093/emboj/cdf474 12198145 PMC125414

[97] Filippakopoulos P, Picaud S, Mangos M, Keates T, Lambert J-P, Barsyte-Lovejoy D, Felletar I, Volkmer R, Müller S, Pawson T, Gingras A-C, Arrowsmith CH, Knapp S. 2012. Histone recognition and large-scale structural analysis of the human bromodomain family. Cell 149:214–231. doi:10.1016/j.cell.2012.02.013 22464331 PMC3326523

[98] Vellmer T, Hartleb L, Fradera Sola A, Kramer S, Meyer-Natus E, Butter F, Janzen CJ. 2022. A novel SNF2 ATPase complex in Trypanosoma brucei with a role in H2A.Z-mediated chromatin remodelling. PLoS Pathog 18:e1010514. doi:10.1371/journal.ppat.1010514 35675371 PMC9236257

[99] Trenaman A, Tinti M, Wall RJ, Horn D. 2024. Post-transcriptional reprogramming by thousands of mRNA untranslated regions in trypanosomes. Nat Commun 15:8113. doi:10.1038/s41467-024-52432-0 39285175 PMC11405848

[100] Wilson K, Uyetake L, Boothroyd J. 1999. Trypanosoma brucei: cis-acting sequences involved in the developmental regulation of PARP expression. Exp Parasitol 91:222–230. doi:10.1006/expr.1998.4366 10072324

[101] Rajan KS, Adler K, Madmoni H, Peleg-Chen D, Cohen-Chalamish S, Doniger T, Galili B, Gerber D, Unger R, Tschudi C, Michaeli S. 2021. Pseudouridines on Trypanosoma brucei mRNAs are developmentally regulated: implications to mRNA stability and protein binding. Mol Microbiol 116:808–826. doi:10.1111/mmi.14774 34165831

[102] Galili-Kostin B, Rajan KS, Ida Ashkenazi Y, Freedman A, Doniger T, Cohen-Chalamish S, Waldman Ben-Asher H, Unger R, Roditi I, Tschudi C, Michaeli S. 2025. TblncRNA-23, a long non-coding RNA transcribed by RNA polymerase I, regulates developmental changes in Trypanosoma brucei. Nat Commun 16:3697. doi:10.1038/s41467-025-58979-w 40251171 PMC12008373

[103] Bard JE, Tylec BL, Dubey AP, Lamb NA, Yergeau DA, Read LK. 2024. Life stage–specific poly(A) site selection regulated by Trypanosoma brucei DRBD18. Proc Natl Acad Sci USA 121:e2403188121. doi:10.1073/pnas.2403188121 38990950 PMC11260167

[104] Radzisheuskaya A, Shlyueva D, Müller I, Helin K. 2016. Optimizing sgRNA position markedly improves the efficiency of CRISPR/dCas9-mediated transcriptional repression. Nucleic Acids Res 44:e141. doi:10.1093/nar/gkw583 27353328 PMC5062975

[105] Sundaresan R, Parameshwaran HP, Yogesha SD, Keilbarth MW, Rajan R. 2017. RNA-independent DNA cleavage activities of Cas9 and Cas12a. Cell Rep 21:3728–3739. doi:10.1016/j.celrep.2017.11.100 29281823 PMC5760271

[106] Dhalluin C, Carlson JE, Zeng L, He C, Aggarwal AK, Zhou M-M, Zhou M-M. 1999. Structure and ligand of a histone acetyltransferase bromodomain. Nature 399:491–496. doi:10.1038/20974 10365964

[107] Mujtaba S, Zeng L, Zhou M-M. 2007. Structure and acetyl-lysine recognition of the bromodomain. Oncogene 26:5521–5527. doi:10.1038/sj.onc.1210618 17694091

[108] Hirumi H, Hirumi K. 1989. Continuous cultivation of Trypanosoma brucei blood stream forms in a medium containing a low concentration of serum protein without feeder cell layers. J Parasitol 75:985–989. doi:10.2307/3282883 2614608

[109] Gunaratne L, Moore H, Albaum N, Casius A, Henderson J, Kessler A, Hegedűsová E, Kulkarni S, Arthur H, Ross RL, Paris Z, Maraia R, Lowe TM, Alfonzo JD. 2025. Key RNA-binding domains in the La protein establish tRNA modification levels in Trypanosoma brucei. Nucleic Acids Res 53:gkaf594. doi:10.1093/nar/gkaf594 40637228 PMC12242769

