# Supplementary material for: The bromodomain protein Bdf3 is sufficient to activate expression of a procyclin gene in bloodstream stage African trypanosomes
Source: mSphere. 2026 Jun 1;11(6):e00021-26. doi: 10.1128/msphere.00021-26 (PMC13317214; doi:10.1128/msphere.00021-26)
Supplement: Supplemental material — Figure S1 and supplemental table legends. [file msphere.00021-26-s0001.docx]

S1 Figure. Representative histograms generated from flow cyometric analysis of parasites transfected with each indicated inducible expression plasmid and guide RNA. Parasites were treated for 3 days with 1µg/ml doxycyclin to induce expression of the indicated protein and the guide RNA. -Dox parasites represent negative controls.

S1 Table. p-values generated from ANOVA statistical test followed by Tukey HSD post-hoc test on flow cytometric data from parasites transfected with inducible dCas9 constructs and guide RNAs treated with 1µg/ml doxycyclin for 48h.

S2 Table. p-values generated from ANOVA statistical test followed by Tukey HSD post-hoc test on flow cytometric data from parasites transfected with inducible dCas9 constructs and guide RNAs treated with 1µg/ml doxycyclin for 72h.
